# Nodal peripheral T-cell lymphomas in the new classification systems

**DOI:** 10.20892/j.issn.2095-3941.2023.0490

**Published:** 2024-02-05

**Authors:** Catalina Amador, Wing C. Chan

**Affiliations:** 1 Department of Pathology, University of Miami Miller School of Medicine, Miami, FL 33136, USA; 2 Department of Pathology, City of Hope National Medical Center, Duarte, CA 91010, USA

Peripheral T-cell lymphomas (PTCLs) encompass a biologically diverse group of non-Hodgkin lymphomas derived from mature T-lymphocytes. Most PTCLs present as nodal diseases and include several subtypes characterized by distinct clinical and pathologic features, and will be the focus of this editorial. The PTCL group presenting as rare distinctive extranodal diseases will not be discussed. While T-cell neoplasms, like B-cell lymphomas, recapitulate stages of normal differentiation, the biology is notably intricate and exhibits remarkable plasticity. Such complexity results in diverse tumor types with numerous distinct entities, each with unique clinical, morphologic, and genetic features. The evolving understanding of PTCLs has led to recent changes in PTCL classification in the 5th edition of the WHO classification (WHO-HAEM5)^1^ and the International Consensus Classification (ICC)^2^ (**Table 1**). This editorial focuses on these nodal PTCL classification changes and discusses the biological underpinnings and clinical implications while exploring the potential impact on future research and patient care.

**Table 1:** Complete nomenclature of nodal T-cell lymphomas in WHO-HAEM4R, ICC2022, and WHO-HAEM5 classifications

WHO-HEAM5	WHO-HEAM4R	ICC 2022
Nodal T-follicular helper (TFH)-cell lymphoma	Nodal lymphomas of T follicular helper (TFH) cell origin	Follicular helper T-cell lymphoma
Angioimmunoblastic-type Follicular-type Not otherwise specified (NOS)	Angioimmunoblastic T-cell lymphoma Follicular T-cell lymphoma Nodal peripheral T-cell lymphoma with T follicular helper phenotype	Angioimmunoblastic type Follicular type Not otherwise specified (NOS)
ALK-positive anaplastic large-cell lymphoma	Anaplastic large cell lymphoma, ALK-positive	Anaplastic large cell lymphoma, ALK positive
ALK-negative anaplastic large-cell lymphoma	Anaplastic large cell lymphoma, ALK-negative	Anaplastic large cell lymphoma, ALK negative
Breast implant-associated anaplastic large cell lymphoma	Breast implant-associated anaplastic large cell lymphoma (provisional entity)	Breast implant-associated anaplastic large cell lymphoma
Peripheral T-cell lymphoma, not otherwise specified	Peripheral T-cell lymphoma, not otherwise specified	Peripheral T-cell lymphoma, NOS
EBV-positive nodal T- and NK-cell lymphoma	Variant of peripheral T-cell lymphoma, not otherwise specified	Primary nodal EBV-positive T-cell/NK cell lymphoma (provisional entity)

## Nodal T follicular helper (TFH) cell lymphoma/follicular helper T-cell lymphoma

The revised WHO 4th edition set an initial framework by introducing the term, nodal lymphomas of TFH cell origin. Under this umbrella, angioimmunoblastic T-cell lymphoma (AITL), follicular T-cell lymphoma, and nodal peripheral T-cell lymphoma with TFH phenotype were grouped together. In its new classification, the ICC 2022 assumes a unifying approach, proposing follicular helper T-cell lymphoma as a unified entity, further subclassified into the following subtypes: angioimmunoblastic type; follicular type; and not otherwise specified (NOS). In parallel, the WHO-HAEM5 opted for nodal TFH cell lymphoma as the family encompassing three related entities. Specific entities were termed, as follows: nodal TFH lymphoma, angioimmunoblastic-type; nodal TFH lymphoma, follicular-type; and nodal TFH lymphoma, NOS.

Despite the subtle differences in terminology, both classifications converge on the fundamental understanding of TFH lymphomas. The classifications acknowledge the commonality of these lymphomas stemming from the observation that AITL exhibits a gene expression profile and immunostaining features similar to normal TFH cells that was subsequently demonstrated in other TFH lymphomas^3–7^. Both classifications recognize the shared mutational landscape of these TFH lymphomas, specifically mutations in *RHOA* G17V and the epigenetic regulators, *TET2* and *DNMT3A*.

The definition of the three TFH lymphomas is similar across both classifications. The angioimmunoblastic and follicular types are established lymphomas with unchanged definitions from WHO-HAEM4R, whereas TFH lymphoma, NOS is less well-defined and more challenging to diagnose. In both classifications, this lymphoma is defined as a mature T-cell lymphoma that does not meet the criteria of any other PTCL but demonstrates strong expression of a minimum of two TFH markers. A minor difference is that the WHO-HAEM5 accepts CD4-negative cases if CD8 is not expressed, while the ICC requires the tumor to be CD4-positive. Both classifications emphasize recognizing the TFH phenotype for accurate diagnosis, suggesting an extended panel of immunohistochemical markers, like CD10, BCL6, PD1/CD279, ICOS, and CXCL13.

The current definition of nodal PTCL with TFH phenotype/nodal TFH lymphoma NOS is based on the immunohistochemical expression of at least two TFH-associated markers. Each marker has a different specificity and sensitivity, and the threshold for positivity has not been defined. In addition, the technical aspects of the assays have not been standardized. Addressing these issues is warranted and further research should be conducted to identify a more robust classifier for this entity.

Similarly, both classifications highlight the importance of mutational profiling, which may facilitate establishing the diagnosis. Preliminary studies suggest that *TET2* mutations are highly prevalent (∼80% in AITL) among all 3 types of TFH lymphomas but are not exclusive to this group. In contrast, the *RHOA* G17V mutation rarely occurs outside these lymphomas^7^. The co-occurrence of *TET2* and *DNMT3A* mutations may be more characteristic of TFH lymphomas. Indeed, there are some reports on the presence of *ITK-SYK* fusion in follicular T-cell lymphomas. Notably, the *IDH2* R172 mutation appears unique to the angioimmunoblastic type^8,9^. Nevertheless, the precise prevalence rates of *TET2, RHOA*, and *DNMT3A* mutations within these three subtypes remain to be defined and the role of mutational profiling in shaping future diagnostic criteria needs additional studies. Additional studies are required to define the mutational profiles of these cases as currently specified.

Recent studies have also underscored the role of clonal hematopoiesis (CH) in the pathogenesis of AITL. CH, manifested by the clonal expansion of a hematopoietic stem/progenitor cell, is caused by acquired somatic mutations in the same epigenetic modulators (i.e., *DNMT3A* or *TET2*) commonly encountered in TFH lymphomas. The mutated clone within the hematopoietic stem cell (HSC) niche exhibits enhanced fitness compared to wild-type cells. The mutation may also influence the differentiation of the progeny of HSCs^10^. Consequently, CH gives rise to identical *TET2* or *DNMT3A* mutation in the both myeloid and lymphoid cells derived from the same mutated stem cell. CH has been detected in many TFHL patients that often had a higher variant allele frequency in the lymphoma than expected from the tumor cell content, suggesting that these TFHL cells and some stromal cells originate from HSCs harboring the CH mutation^11^. The presence of additional, private mutations (e.g., *RHOA* and/or *IDH2*) in the TFH lymphoma component, but not in the corresponding CH, indicates that the CH clone may initiate the TFHL oncogenesis, but further evolution of the T-cells within a suitable microenvironment is needed for neoplastic transformation.

## Anaplastic large cell lymphoma (ALCL)

Systemic ALCLs usually present as advanced disease with nodal involvement and are therefore discussed herein, although extranodal involvement is also frequent. Both remain broadly consistent with the 2017 WHO classification, recognizing ALK-positive and -negative ALCLs as distinct entities. BIA-ALCLs are distinct from other ALK-neg ALCLs and are no longer a provisional variant in either of the two classifications. Notably, WHO-HAEM5 does not include provisional entities. In both classifications, primary cutaneous ALCLs are grouped separately, indicating the close relationship to primary cutaneous T-cell lymphoid proliferations and lymphomas. While all ALCL entities have an anaplastic morphology with hallmark cells and very strong, uniform CD30 expression, each subtype demonstrates unique molecular features.

No major changes have been described in ALK-positive ALCL, which is defined by the presence of ALK fusion proteins due to *ALK* rearrangements with different partner genes. The resulting ALK fusion protein leads to inappropriate and constitutive activation of the ALK kinase function. This process initiates a cascade of cellular signaling pathways, prominently including PLC-γ, PI3K/AKT, Cdc42/Rac1, JNK/cJun, RAS/ERK/MAPK, mTOR, JAK/STAT3, and the STAT5B pathways.

While ALK-negative ALCL has often been considered a single entity in prior classifications, emerging data underscore the molecular heterogeneity. Like ALK-positive ALCL, there is activation of the JAK-STAT3 pathway in approximately two-thirds of ALK-negative ALCLs. This activation is usually secondary to *JAK1*, *JAK3*, or *STAT3* mutations or rearrangements involving *TYK2*, *ROS1*, or *FRK*^12,13^. In contrast, approximately 30% of ALK-negative ALCLs display rearrangements at the *DUSP22* locus (6p25.3) and exhibit no JAK-STAT3 pathway activation or a DNA hypomethylation state^14^. The WHO-HAEM5 retains ALK-negative ALCL as a unified entity but acknowledges the molecular diversity. Conversely, the ICC recognizes cases with DUSP22 rearragements as a distinct genetic subtype of ALCL (ALK-negative) that is characterized by unique pathologic and molecular features, including a unique signature gene expression, DNA hypomethylation, recurrent *MSC* mutations, and characteristic morphologic features, such as small doughnut-shaped cells and LEF1 expression and the absence of cytotoxic markers^12–14^. A small subset (<10%) of ALK-negative ALCLs have *TP63* gene rearrangements, which have been associated with aggressive clinical behavior and poor outcomes^14^.

## Peripheral T-cell lymphoma, not otherwise specified (PTCL-NOS)

The ICC and WHO-HAEM5 classifications agree on the definition of PTCL-NOS as a mature T-cell lymphoma that does not meet the criteria for any specific PTCL subtype. The classification has evolved with the exclusion of EBV-positive T/NK-cell lymphoma primarily involving nodes. Once included as a PTCL-NOS variant, EBV-positive T/NK-cell lymphoma is now acknowledged as its own entity due to distinct clinicopathologic and molecular characteristics^15,16^. Similarly, excluding cases that express two or more TFH markers is critical because these PTCL-NOS are now regarded as a type of TFH lymphoma.

Gene expression profiling has highlighted two main molecular subgroups within PTCL-NOS (PTCL-GATA3 and PTCL-TBX21) each bearing unique morphologic, molecular, and clinical features^6,17,18^ (**Table 2**). PTCL-GATA3 is linked to a worse prognosis and exhibits increased genomic complexity with high-frequency deletions of many tumor suppressor genes, such as *TP53, PTEN, CDKN2A*, and *PRDM1*. At the same time, PTCL-TBX21 is characterized by mutations in epigenetic regulators, suggesting distinct oncogenic pathway involvement^6,19^. An immunohistochemical algorithm has been proposed as an alternative to molecular classification in routine clinical practice^18^. Moreover, a digital gene expression classifier for nodal T-cell lymphomas has been introduced, showing the potential to distinguish between PTCL-GATA3 and PTCL-TBX21 using paraffin-embedded tissues^20^.

**Table 2:** Characteristics of two major molecular subgroups of PTCL-NOS identified by gene expression profiling studies

PTCL-NOS, GATA3	PTCL-NOS, TBX21
Overexpression of GATA3 and related genes Upregulation of mTOR/PI3K pathways Greater genomic complexity Deletions & mutations of many tumor suppressor genes Worse prognosis Minimal tumor microenvironment; more monomorphic morphology CD4+/CD8- or CD4-/CD8- without expression of cytotoxic antigens	Overexpression of TBX21 and related genes Upregulation of interferon and NF-κB pathways Lesser genomic complexity Mutations in epigenetic regulators Better prognosis Prominent tumor microenvironment; more polymorphic morphology Variable expression of CD4 and CD8; subset shows cytotoxic antigen expression

PTCLs with a cytotoxic gene expression signature were observed and later linked to the PTCL-TBX21 subgroup. These cases appeared to have an unfavorable prognosis compared with the other TBX21 cases^17,19,21^. However, the significance of CD4/8 and cytotoxic marker expression in PTCL-NOS diagnosis has yet to be completely elucidated. Further studies are needed to determine the most reliable cytotoxic T-cell differentiation/identity indicators and how best to implement them in the clinical setting. This would pave the way for defining cytotoxic PTCL as a separate entity.

PTCL-NOS is often portrayed as a group of lymphomas with an inferior prognosis and limited therapeutic options. As discussed above, PTCL-NOS heterogeneity is well-delineated, but additional molecular studies and functional analyses are required to clearly define these entities, refine diagnostic criteria, and open avenues for advanced targeted treatments.

## Primary nodal EBV-positive T-/NK-cell lymphoma/ EBV-positive nodal T- and NK-cell lymphoma

According to the previous WHO-HAEM4R classification, tumors with EBV-encoded RNAs (EBERs) present in most neoplastic cells and primary nodal presentation were classified as primary nodal EBV+ T/NK-cell lymphoma and listed as a variant of PTCL, NOS. However, in the recent WHO-HAEM5 and ICC classifications, primary nodal EBV+ T/NK-cell lymphoma has been promoted to a new entity status. The WHO-HAEM5 recognizes it as a distinct entity, designating it as EBV-positive nodal T- and NK-cell lymphoma. The ICC categorizes it as a provisional entity, under the designation of primary nodal EBV+ T/NK-cell lymphoma. Currently, in both classifications these tumors are considered distinct from nodal PTCL-NOS with expression of cytotoxic markers that are EBV-negative and extranodal EBV-positive NK-/T-cell lymphomas.

This entity is now identified as a unique group, typically presenting with systemic lymphadenopathy without nasalor nasopharyngeal involvement. A primary nodal EBV+ T/NK-cell lymphoma mostly consists of cytotoxic CD8+ T-cell tumors but also includes NK-cell derived tumors, which are distinct from extranodal NK/T-cell lymphomas (ENKTCLs), in which NK-cell tumors predominate. Primary nodal EBV+ T/NK-cell lymphomas are often detected in immunocompromised patients with expression of proteins related to immune evasion, such as PD-L1^15^. Due to the phenotypic similarity to ENKTCL, the classification underscores the need for cautious diagnosis because ENKTCL occasionally affects lymph nodes secondarily.

While these lymphomas are frequently analyzed as a collective group, without differentiating between NK cell-derived and T-lineage-derived cases, such an approach might obscure the genetic, molecular, and clinical distinctions between the subgroups. It is imperative for future research to address these issues.

## Conclusions

The WHO-HAEM5 and ICC2022 classification systems have introduced important advances to the classification of nodal mature T-cell neoplasms, enhancing our comprehension and leading to a more precise diagnosis of these complex lymphomas. Despite these significant improvements, the continuous understanding of nodal T-cell lymphomas should remain an ongoing scientific endeavor. While the differences between the proposed classifications in this area are minor and mainly revolve around terminology, it is crucial for future revisions to unify the terminology to establish a common language, which is critical for both effective scientific communication and continued advances in the field. While future research should be directed towards a deeper exploration of the molecular underpinnings of these lymphomas and the discovery of potential novel therapeutic targets, a more immediate focus should be to define more clearly the diagnostic boundaries between different entities, and in particular, nodal TFH cell lymphoma/AITL/PTCL-NOS and cytotoxic T-cell lymphoma/helper PTCL-TBX21.
